# A82 PREVALENCE AND NON-INVASIVE SCREENING FOR CYSTIC FIBROSIS RELATED LIVER DISEASE IN A COHORT FOLLOWED AT A CYSTIC FIBROSIS REFERENCE CENTER

**DOI:** 10.1093/jcag/gwac036.082

**Published:** 2023-03-07

**Authors:** D E Coman, C Vincent, A Lavoie, M Bilodeau, J Hercun

**Affiliations:** 1 Internal Medicine, CHUM; 2 Université de Montréal; 3 Hepatology; 4 Pneumology, CHUM, Montreal; 5 Université de Montréal, Montréal, Canada

## Abstract

**Background:**

The reported prevalence of cystic fibrosis (CF) related liver disease (CFLD) varies widely based on diagnostic criteria but reaches up to 40% in some cohorts. Furthermore, its clinical impact is significant as hepatic involvement is the 3^rd^ leading cause of mortality in CF patients and is associated with a lower life expectancy. Due to the heterogeneous clinical presentation of CFLD, clear diagnostic criteria and non-invasive assessment methods are lacking.

**Purpose:**

This study aimed to measure the prevalence of CFLD in a cohort of patients followed at a tertiary CF center.

**Method:**

The files of all patients followed at the CF clinic of the Centre hospitalier de l’Université de Montréal in 2021 were retrospectively reviewed. Imaging reports, histopathology, laboratory values and transient elastography results were assessed. The NIH criteria were used to define CFLD through either presence of one major criteria (abnormal imaging) or two minor criteria (persistently abnormal lab values, hepatosplenomegaly, or transient elastography ≥ 7 kPa).

**Result(s):**

358 patients were included in our study, 56% male with a median age of 36 years. While mean liver tests were within normal limits (ALT 24 U/L, AST 26 U/L, ALP 96 U/L, GGT 29 U/L), 182 patients (51%) had at least one episode of abnormal liver function tests (LFTs), and 85 patients (24%) had persistently abnormal LFTs. CFLD was present in 42 patients (12%), with 39 patients presenting major criteria and 3 patients presenting minor criteria. In addition, 67 patients (19%) had solely hepatic steatosis. Furthermore, clinically significant portal hypertension and esophageal varices were detected in 50% of patients with CFLD who underwent upper endoscopy (n=20). Median transient elastography value was 5.4 kPa (interquartile range 4.25 kPa) in the 51 patients with exam results, and 32% had values ≥ 7 kPa. Fibroscan values correlated well with the presence of major criteria, with an area under the curve (AUROC) of 0.80 (0.68-0.92, p=0.0007), while non-invasive serological markers did not perform as well (Fib4: AUROC 0.70 (0.59-0.81) and APRI: AUROC 0.69 (0.59-0.80)).

**Conclusion(s):**

The overall prevalence of CFLD, including hepatic steatosis, was 31% in this cohort. Prompt recognition is important in clinical care in order to prevent hepatic complications from cirrhosis and portal hypertension. The use of Fibroscan seems promising for detecting CFLD.

**Please acknowledge all funding agencies by checking the applicable boxes below:**

None

**Disclosure of Interest:**

None Declared

